# Phylogenetic composition and distribution of picoeukaryotes in the hypoxic northwestern coast of the Gulf of Mexico

**DOI:** 10.1002/mbo3.57

**Published:** 2012-12-27

**Authors:** Emma Rocke, Hongmei Jing, Hongbin Liu

**Affiliations:** Division of Life Science, Hong Kong University of Science and TechnologyClear Water Bay, Kowloon, Hong Kong

**Keywords:** Dinoflagellates, hypoxia, MALV I and II, picoeukaryotes, rDNA environmental sequences

## Abstract

Coastal marine hypoxic, or low-oxygen, episodes are an increasing worldwide phenomenon, but its effect on the microbial community is virtually unknown by far. In this study, the community structure and phylogeny of picoeukaryotes in the Gulf of Mexico, which are exposed to severe hypoxia in these areas was explored through a clone library approach. Both oxic surface waters and suboxic bottom waters were collected in August 2010 from three representative stations on the inner Louisiana shelf near the Atchafalaya and Mississippi River plumes. The bottom waters of the two more western stations were much more hypoxic in comparison to those of the station closest to the Mississippi River plume, which were only moderately hypoxic. A phylogenetic analysis of a total 175 sequences, generated from six 18S rDNA clone libraries, demonstrated a clear dominance of parasitic dinoflagellates from Marine alveolate clades I and II in all hypoxic waters as well as in the surface layer at the more western station closest to the Atchafalaya River plume. Species diversity was significantly higher at the most hypoxic sites, and many novel species were present among the dinoflagellate and stramenopile clades. We concluded that hypoxia in the Gulf of Mexico causes a significant shift in picoeukaryote communities, and that hypoxia may cause a shift in microbial food webs from grazing to parasitism.

## Introduction

The worldwide frequency of estuarial and coastal hypoxia has increased from ∼20 to ≥400 sites over the past century due to anthropogenic loading of nutrients in estuarine environments (Diaz and Rosenberg 2008; Rabalais et al. 2010; Zhang et al. 2010). Nutrient overload usually due to agricultural activities in these systems causes massive and often toxic algal blooms, which upon dying become a source for aerobic decomposition, absorbing oxygen from the underlying water layers. Coastal ecosystems all over the world are experiencing such conditions (Diaz and Rosenberg 2008) as worldwide eutrophication impacts increase and temperatures rise, leading to increased respiratory demand of organisms and decreased oxygen solubility (Helm et al. 2011). The Mississippi River discharge directly impacts the Gulf of Mexico (GOM) ecosystem, and as such is considered symptomatic of these many similar situations spreading worldwide.

The inner Louisiana shelf has suffered from seasonal hypoxia since the early 1990s. Freshwater discharge from the Mississippi River system along with seasonal atmospheric warming control the stratification that is needed to maintain these hypoxic episodes. The Mississippi River system discharges ∼30% through the Atchafalaya River delta (∼7300 m^3^ sec^−1^) and ∼70% through the Mississippi birdfoot delta (1.7 × 10^4^ m^3^ sec^−1^). Of the total Mississippi River discharge, 53% flows west onto the Louisiana shelf and all of the Atchafalaya River discharge flows to the west (Rabalais and Turner 2001). Maximum stratification during the summer is maintained due to the strength of this river discharge, wind mixing, regional circulation and air–sea heat-exchange processes (Rabalais and Turner 2001), providing perfect conditions for hypoxic episodes.

Picoeukaryotes are an incredibly diverse assemblage of organisms, each species able to thrive in their own unique niche (Romari et al. 2004; Countway et al. 2007; Nolte et al. 2010). Community studies to date have shown an incredible amount of undescribed, “rare” taxa. These “rare” taxa normally present in relatively low numbers, combined with a group of dominant taxa (Schloss and Handelsman 2004; Countway et al. 2005; Epstein and Lopez-Garcia 2008). It is hypothesized that these “rare” taxa represent an adaptable community able to take over in changing environmental conditions and are able to maintain biogeochemical processes during stressful environmental episodes such as hypoxia (Caron and Countway 2009). This structure could therefore act as a biological buffer to the impact of such episodes by maintaining important ecosystem processes. It is with this viewpoint that the distribution of some rarer taxa in this study may provide further insight into the response of the marine microbial diversity to stresses such as hypoxia. Attention should be paid to the methods used in this study, however, which cannot compare to high-throughput sequencing methods.

Studies such as Fenchel and Finlay (2008) have found that some picoeukaryotes thrive at specific oxygen tensions, creating their own niche among competitors with similar living and feeding habits. As many species feed on bacteria, low-O_2_ waters could act as a cue for feeding, as bacterial production tends to be elevated at or below the oxycline (Fenchel et al. 1990; Fenchel and Finlay 2008). Oxygen uptake is also known to be limited by molecular diffusion at low O_2_ tensions. As such, respiration rates can be related to cell size and volume. This creates a special niche for small aerobic organisms such as picoeukaryotes in microaerobic conditions. Phylogenetically, picoeukaryote distribution in hypoxic/anoxic waters could provide valuable insight into the early evolution of eukaryotes (Cavalier-Smith 2002), as well as hypoxic shifts in energy pathways and elemental flow in marine food webs.

Very few studies to date have focused on picoeukaryote survival and distribution in hypoxic waters (Fenchel and Finlay 2008). Along with the development and application of molecular tools to study microbial species distribution, several clone library studies focusing on picoeukaryotes in low-oxygen environments so far have revealed substantially rich species diversity (Dawson and Pace 2002; Behnke et al. 2006; Stoeck et al. 2006; Zuendorf et al. 2008). As hypoxia is well documented to cause habitat compression, loss of fauna, and ecosystem energy diversion into microbial pathways (Hoback and Barnhart 1996; Diaz and Rosenberg 2008), further insight into picoeukaryote species distribution in these habitats is crucial. The goal of this study therefore is to compare the picoeukaryote species distribution in oxic surface waters versus hypoxic bottom waters, and to describe the effects of hypoxia on picoeukaryotes among samples taken from the hypoxic Louisiana shelf during the summer of 2010 using a clone library approach.

## Materials and Methods

### Sample collection

**Figure 1:**
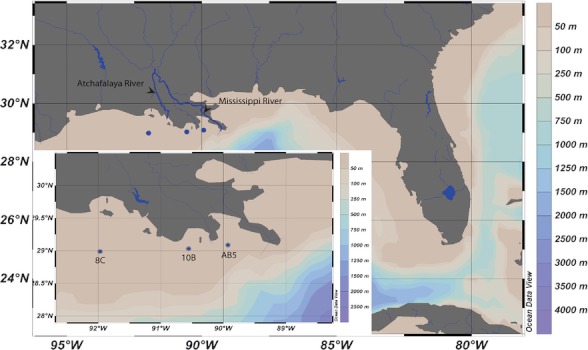
Geographic location of the three stations from the inner Louisiana Shelf in the northwestern Gulf of Mexico. Station AB5: 29**°**02′N, 90**°**33′W; Station 10B: 29**°**05′N, 89°56′W; Station 8C: 28**°**59′N, 19°58′W.

**Figure 2:**
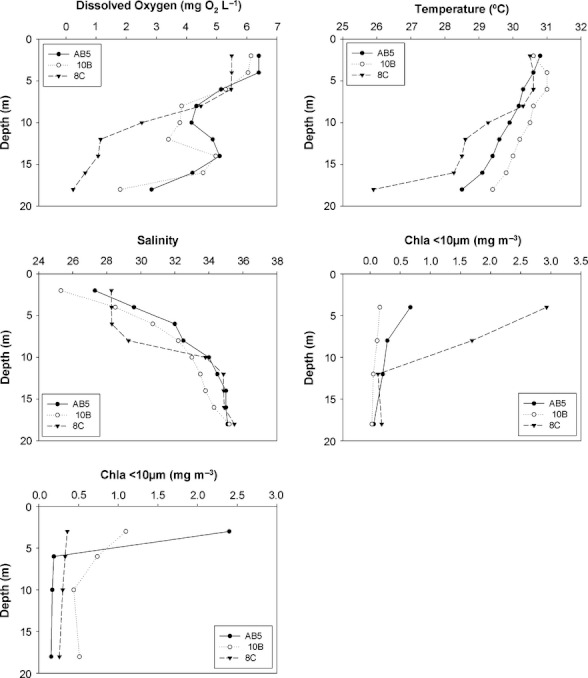
Depth profiles of dissolved oxygen (O_2_), salinity, temperature, and chlorophyll *a* for all three stations sampled.

The three stations in this study were located just south of Barataria Bay (AB5: 29°02′N, 90°33′W), Terrebonne Bay (10B: 29°05′N, 89°56′W), and Atchafalaya Bay (8C: 28°59′N, 91°58′W) (Fig. 1). Samples were collected from the surface and subpycnocline layers at these three stations on the inner Louisiana shelf (Fig. 1). Sampling was carried out on 15–18 August 2010. Seawater temperature, salinity, and dissolved oxygen were recorded by a Seabird CTD profiler. Chlorophyll *a* samples were extracted in two size fractions (>10 μm, <10 μm) and analyzed by the methods described in Liu and Dagg (2003). Each water sample (125–350 mL) was filtered first through a 3-μm and then through a 0.22-μm pore-sized polysulfone/polycarbonate filter (GE Water & Process Technologies, Feasterville-Trevose, PA) using a vacuum pump. The filter was then transferred to a 1.5-mL tube (Axygen, Union City, CA), immediately frozen, and stored at −80°C until DNA extraction (Fig. 2).

### DNA extraction and amplification

DNA was extracted from the 0.22-μm filters using a modified phenol: chloroform extraction and alcohol precipitation procedure (Boström et al. 2004). The 18S rDNA fragments were amplified by polymerase chain reaction (PCR) using the oligonucleotide markers Euk328f (5′-ACC TGG TTG ATC CTG CCA G-3′) and EUK329r (5′-TGA TCC TTC YGC AGG TTC AC-3′) (Moon-van der Staay et al. 2001). The PCR reaction was carried out with a 20-μL master mix containing 2.5 μL of 10× buffer, 1 μL of MgCl_2_ (25 mmol/L) and dNTPs (5 mmol/L), 0.5 μL of BSA (bovine serum albumin) and forward and reverse primer (10 μmol/L), 0.1 μL of Taq polymerase (Invitrogen, Carlsbad, CA), and 2 μL of DNA template. The PCR program consisted of an initial incubation step at 94°C for 5 min, followed by 35 cycles with a denaturation step at 94°C for 1 min, an annealing step at 59°C for 2 min, and then an extension step at 72°C for 3 min. These cycles were followed by a final extension step at 72°C for 10 min. Amplifications were verified on a 0.8% agar gel stained with ethidium bromide.

### Clone library construction and sequencing

PCR-amplified extracts were purified using the GE Illustra™ GFX™ PCR (Buckinghamshire, UK) DNA and Gel band Purification Kit. After purification of the PCR products, they were cloned into Escherichia coli using the pCR2.1-TOPO TA vector system (Invitrogen®) following the manufacturer's instructions. Screening for a positive insert was done so through PCR amplification of white colonies using the vector-specific M13 primers. Positive clones were then sent to BGI for partial sequencing (∼1800 bp) using the M13 forward and reverse specific primers. Sequences were assembled using Bioedit 7.0.9 (Hall 1999).

### Phylogenetic and statistical analysis

**Figure 3:**
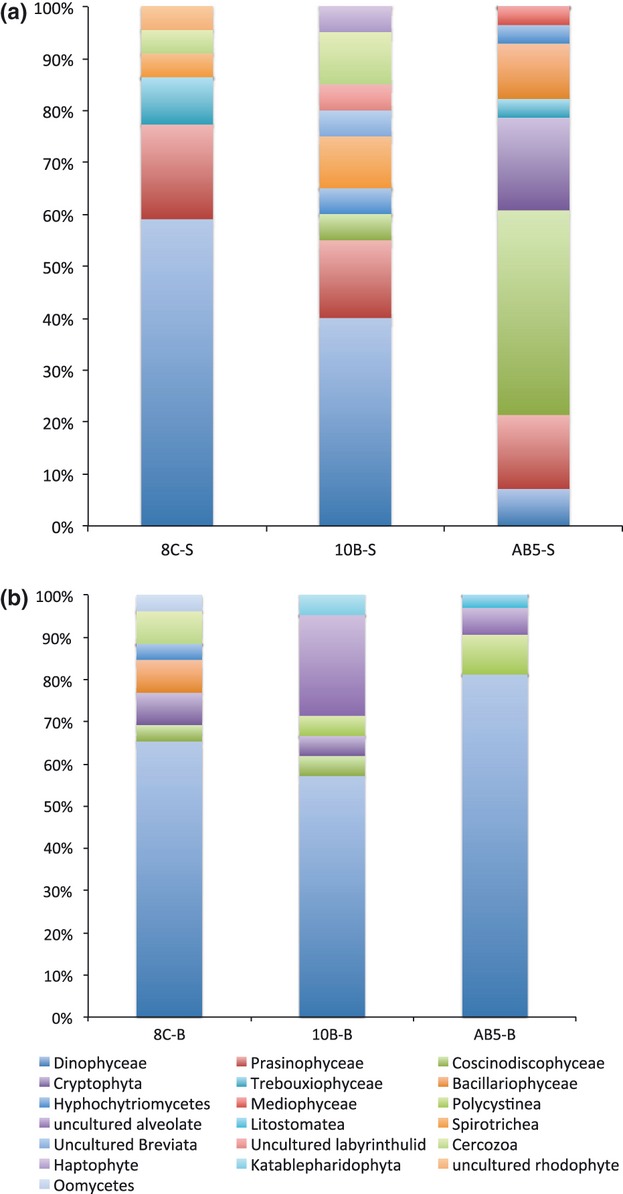
Picoeukaryote community compositions at the class taxonomic level for the six samples collected from the three stations based on the 18S rDNA sequence libraries. (a) Surface waters; (b) bottom waters.

A BLAST search was conducted using the Genbank database (http://blast.ncbi.nlm.nih.gov). As sequences could not always be identified down to the species level, they are presented by class (Fig. 3). For all six clone libraries, sequences were aligned using CLUSTALW (Thompson et al. 1994). The phylogenetic tree along with distance and bootstrap analysis were performed using the PHYLIP program (Felsenstein 1989). The sequences were sorted into operational taxonomic units (OTUs) using the MOTHUR program (Schloss et al. 2009) using a 99% sequence similarity. Shannon and Simpson species diversity and evenness indices were then calculated from these resulting OTUs using MOTHUR. A distance matrix of aligned sequences was generated using DNADIST (Felsenstein 1989). A principle coordinate analysis (PCoA) plot and corresponding analysis of molecular variance (AMOVA) was calculated using MOTHUR in order to test the significance of the plot's molecular variation between hypoxic and oxic habitats.

### Nucleotide sequence accession numbers

The 18S rDNA gene sequences generated by this study have been filed in GenBank under the accession numbers JF790975–JF791112.

## Results

### Study site

The observed oxygen levels remained high at the surface for all three stations, measuring at 6.4, 6.2, and 5.3 mg O_2_ mL^−1^ for stations AB5, 10B, and 8C, respectively (Table 1). Subpycnocline values differed however. The oxygen level at station AB5 was 3.2 mg O_2_ L^−1^. However, stations 8C and 10B were hypoxic with oxygen values of 0.3 and 1.18 mg O_2_ L^−1^, respectively. Temperature remained fairly constant at around 30°C at the surface of each station, and 27–28°C at the subpycnocline layer. The pycnocline of station 8C was slightly colder at 26.3°C. Salinity was much lower at the surface compared with the bottom waters of each station.

**Table 1:** Environmental parameters at the surface and bottom waters of each sampling station

	AB5-S (3 m)	AB5-B (18 m)	10B-S (2 m)	10B-B (20 m)	8C-S (4 m)	8C-B (18 m)
Dissolved oxygen (mg O_2_ L^−1^)	6.4	3.2	6.2	1.18	5.3	0.3
Temperature (°C)	31	28.5	31	28	30.5	26
Salinity	27.3	35	26.3	35.4	27.1	35.5

Chlorophyll *a* concentrations were >3 mg m^−3^ at surface layer of stations AB5 and 8C, but the former contained higher concentrations of the <10-μm fraction, while the latter contained higher concentrations of the >10-μm fraction (Fig. 2). Station 10B had a lower chlorophyll concentration (1.26 mg m^−3^ at 2 m) and this was also mainly from the <10-μm fraction.

### Clone library composition

Surface waters of station AB5, located closest to the Mississippi River mouth, just south of Barataria Bay showed a dominance of the diatom Coscinodiscophyceae (39%), with the remaining classes consisting of Cryptophytes (18%), Prasinophytes (14%), and Bacillariophytes (11%). Dinoflagellates dominated the surface waters of both station 10B (40%) and 8C (59%). Prasinophytes also occupied a significant part of station 10B and 8C surface waters (15% and 18%, respectively).

**Figure 4:**
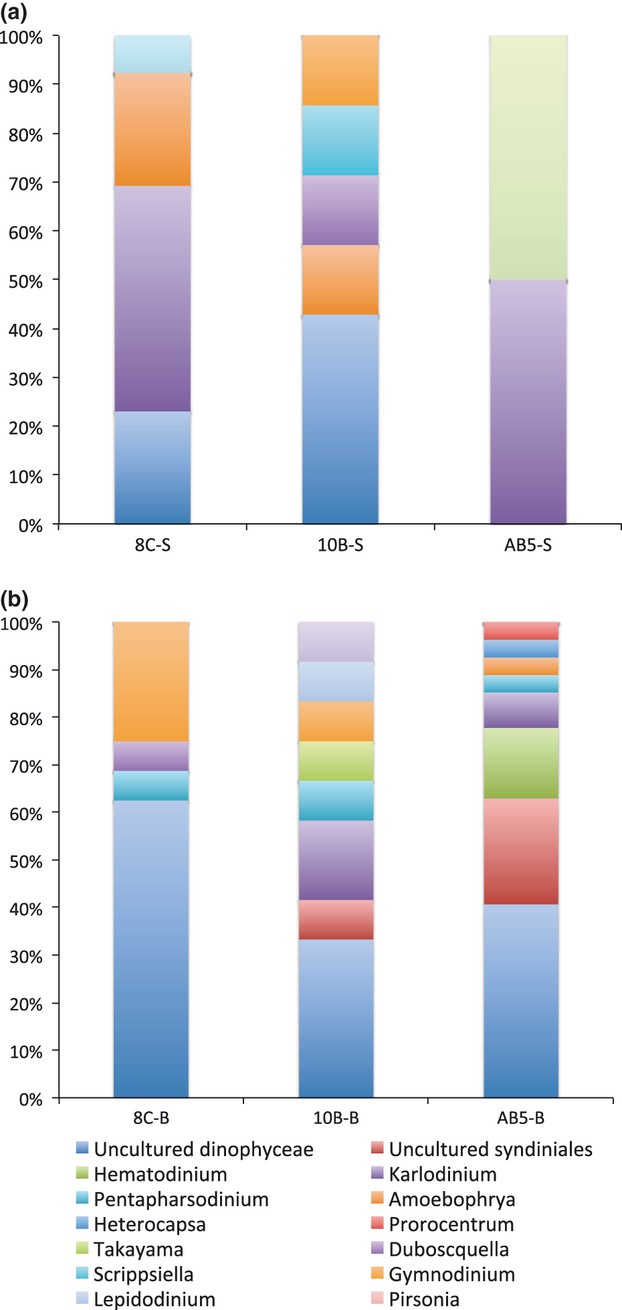
Community composition of Dinoflagellates by genera at the surface and bottom waters at each station. (a) Surface waters; (b) bottom waters.

Bottom waters of station AB5 consisted of 81% dinoflagellates, the rest consisting of Polycystinea (9%) and uncultured alveolates (6%). Dinoflagellates also dominated by far in the other two bottom-water clone libraries, consisting of 57% and 65% of stations 10B and 8C bottom waters, respectively. Other dominant bottom-water classes include uncultured alveolates in the subpycnocline sample of station 10B (24%) (Fig. 3). Species diversity, the highest of which was found in hypoxic clone libraries is not immediately visible in Figure 3 as the dinoflagellate class contains unclassified or unknown OTUs, which are further illustrated in Figure 4 and in the phylogenetic analysis.

Dinoflagellate-affiliated OTUs were prominent in all samples except for the surface waters of station AB5, and so this class was further subdivided into family or genus if possible (Fig. 4). Of all subpycnocline OTUs, 83% were classified as dinoflagellates. Surface samples consisted of uncultured dinoflagellates, *Karlodinium* and *Amoebophyra* at stations 8C and 10B. Very few dinoflagellates occupied the surface waters of station AB5, and so these results are negligible. Uncultured dinoflagellates, Syndiniales and Gymnodinium mostly occupied bottom samples. These OTUs were more clearly distinguished after phylogenetic analysis.

**Figure 5:**
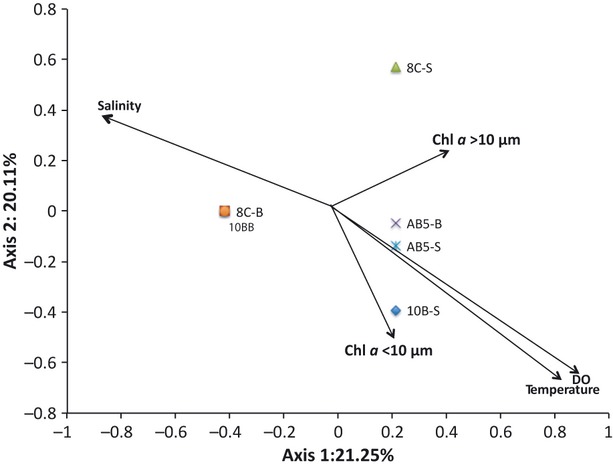
PCoA plot of all the 18S rDNA gene sequences retrieved from this study at the 98% sequence-identity threshold. Arrows indicate Eigenvectors with its length indicating Spearman correlation between environmental variables and phylogenetic composition of each clone library. Clone libraries are represented in the biplot by different symbols. Axis 1 and 2 represent 21.25% and 20.11% of the total variance in operational taxonomic unit (OTU) distribution, respectively.

A PCoA plot of all six clone libraries showed definitive grouping of hypoxic bottom samples apart from all other samples, and eigenvectors confirmed that oxygen, temperature, and salinity have the strongest influence on picoeukaryote distribution (Fig. 5, *P* < 0.01). Chl *a* showed no significant correlation with samples; however, they do appear vaguely associated to AB5S (Chl *a* < 10 μm) and 8CS (Chl *a* > 10 μm), both of whom contained higher Chl *a* concentrations of these size classes. AMOVA analysis confirmed that bottom hypoxic sample data points in the PCoA plot clustered away significantly from surface water data points (*P* < 0.001). On the other hand, species compositions in the surface and bottom layers of AB5 were very similar as they were located together in the PCoA plot (Fig. 5). Species diversity, as indicated by Shannon's diversity index was higher in the subpycnocline hypoxic samples from station 8C and 10B (Table 2). Species diversity for each sample was found to be significantly different between every station and depth (unifrac significance, *P* = 0.001).

**Table 2:** Composition and diversity of the six clone libraries with samples collected from the three stations at 97% sequence-similarity threshold

Sample	Number of clones	Number of OTU	*H*′	*D*_S_	*J*′	ACE
AB5-S	26	15	2.99	0.0015	0.98	1010
AB5-B	33	12	2.39	0.009	0.99	484
10B-S	27	14	2.83	0.0015	0.98	111
10B-B	25	12	3.09	0.001	0.98	1039
8C-S	25	10	2.65	0.035	0.96	159
8C-B	30	17	3.17	0.001	0.98	1452

Shannon (*H*′), Simpson (*D*_**S**_), evenness (*J*′), and abundance-based coverage estimation (ACE) indexes. OTU, operational taxonomic unit.

### Phylogenetic analysis

**Figure 6:**
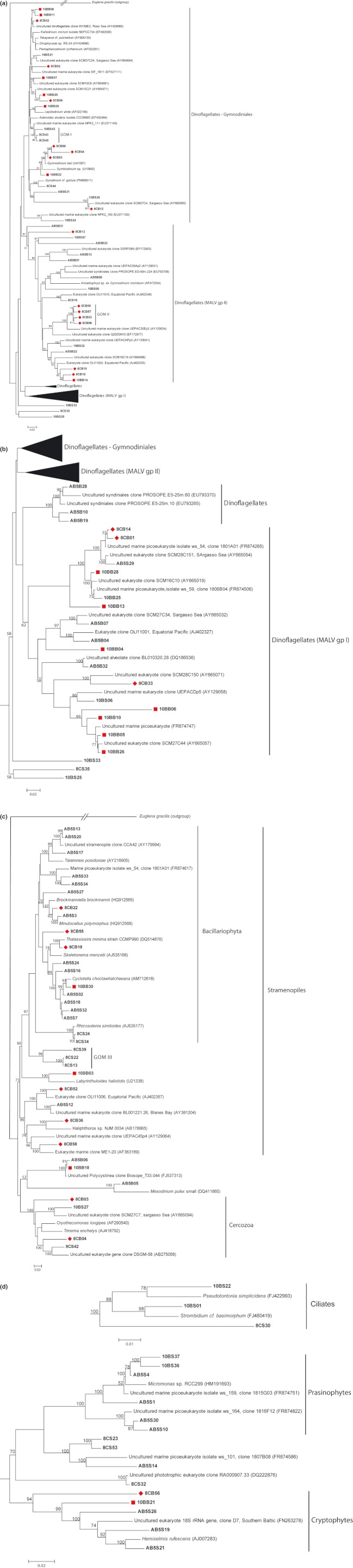
Phylogenetic relationships of picoeukaryotes based on maximum likelihood analysis of partial 18S rDNA gene sequences from all six clone libraries from the Gulf of Mexico. The scale bar indicates 0.02 nucleotide changes per position. Bootstrap values above 50 were indicated on the nodes. The outgroup is *Euglena gracilis*. Red icons represent clones collected under hypoxic conditions. (a) Gymodiniales and MALV II dinoflagellate clades; (b) MALV I dinoflagellate clade; (c) stramenopile and cercozoan clades; (d) Prasinophyte, cryptophyte, and ciliate clades.

Dinoflagellate OTUs clustered into three distinct clades (Fig. 6). The Gymnodiniales clade represents heterotrophic dinoflagellates of the order Gymnodiniales, closely related to the species *Gymnodinium*. Three OTUs from 8C surface waters clustered here, two of which formed the unique cluster GOM I within the *Gymnodinium* II clade. Six OTUs came from 10B bottom waters, and the remaining seven OTUs in this cluster came from the hypoxic 8C bottom-water sample. The second dinoflagellate clade clustered with the parasitic Marine alveolate (MALV) group II. One OTU of a total of 18 belonged to station 10B bottom waters, six to station AB5 bottom waters, six to 8C bottom waters, and three to the surface waters of station 10B. A unique, exclusively 8CB cluster, GOM II formed here. The final dinoflagellate clade was from MALV group I. This clade consisted of three OTUs out of 16 from AB5 bottom waters, three from 8C bottom waters, one OTU from station 10B surface waters, and eight OTUs from 10B bottom waters.

Twenty-seven OTUs clustered with the Stramenopiles. AB5S OTUs dominated the stramenopile clade, with 14 OTUs overall, followed by 8CB (six OTUs), 8CS (five OTUs), and 10BB (two OTUs). Thirteen AB5S, three 8CB, two 8CS, and one 10BB OTUs clustered with Bacillariophyta, or diatoms. The remaining 8CS OTUs formed a unique cluster next to the diatoms (GOM III). The rest of the hypoxic 8CB and 10BB OTUs clustered with Labyrinthulida and Oomycetes subclasses.

The Cercozoan cluster consisted of OTUs from 8CB (2), 10BS (1), and 8CS (1). Prasinophytes included OTUs from AB5S (4) and 10BS (2). Cryptophytes were dominated by AB5S (three OTUs), then 8CB and 10BB (one OTU each). Finally, ciliates clustered with three OTUs, from 10BS (2) and 8CS (1).

## Discussion

The GOM represents one of the longest studied human-induced estuarial hypoxic or “dead” zones (Rabalais and Turner 2001), and as such represents the ideal location to study its effects on the microbial food web. To date, no study has sampled and compared picoeukaryote communities from oxic versus anthropogenically hypoxic conditions in subtropical waters. The sites focused on in this study represent the ideal situation in which to do so, as all measured physical and environmental parameters remained constant across all sites and depths except for dissolved oxygen concentration and chlorophyll, the latter of which was only significantly higher for the oxic surface samples at stations AB5 and 8C. The data analyzed will therefore be presented and interpreted with respect to their oxygen concentration.

Consistent with previous studies focusing on picoeukaryote diversity in general (Massana et al. 2004; Romari et al. 2004; Lovejoy et al. 2006; Medlin et al. 2006; Not et al. 2007; Vaulot et al. 2008), the results from this study showed significantly distinct species assemblages at each station and depth. The highest diversity can be found in the most hypoxic samples. Such a trend is common in the literature (Stoeck et al. 2010; Edgcomb et al. 2011; Orsi et al. 2012). In addition to increased species diversity, phylogenetic results show a dominance of parasitic species in hypoxic waters, as well as a novel dinoflagellate clade. This could suggest a significant change in trophic links in hypoxic habitats.

### Hypoxic bottom samples

When oxic and hypoxic samples were compared in this study, the hypoxic samples 8C_B and 10B_B showed a significantly higher species diversity and richness compared with oxic samples. These samples also clustered separately from all other samples in the PCoA analysis, showing a significantly different community structure in the hypoxic waters of the GOM (AMOVA, *P* < 0.001). This suggests that the picoeukaryote communities in the hypoxic samples contain more rare or unknown taxa, and they could shift in a unique way when exposed to hypoxic conditions, acting as a biological buffer (Caron and Countway 2009). Interpretation of the phylogenetic analysis in this study also seems to point in this direction. It is important to note, however, that as this study uses a clone library approach with one time sampling only, we are unable to provide conclusive evidence to this theory at this time. What we may perceive as our “rare” sequences could in fact become more abundant should another method such as pyrosequencing be used. With respect to the overall protist community studied to date, this study may conclude, however, that BLAST results from hypoxic waters affiliated with many OTUs from the other few studies focusing on protists in hypoxic to anoxic waters (Stoeck and Epstein 2003; Stoeck et al. 2010; Edgcomb et al. 2011; Orsi et al. 2012). This study also finds many novel unclassified dinoflagellates from the MALV I and II clades, which suggests the beginning of a shift in ecosystem energy flow from grazing to parasitism. We were also able to identify three novel clusters unrelated to any BLAST search result, one of which affiliated with the MALV II clade.

The majority of the hypoxic samples clustered with the dinoflagellates. These sequences were evenly distributed between heterotrophic dinoflagellates from the order Gymnodiniales (42%) and the novel marine alveolate lineages (MALV) I and II (58%) (López-García et al. 2001; Moon-van der Staay et al. 2001), also named Syndiniales groups I and II (Guillou et al. 2008). Hypoxic gymnodiniales OTUs split evenly between stations 10B and 8C. Gymnodiniales, an order of nonthecate dinoflagellates can be either photosynthetic or heterotrophic and are most commonly known for their bloom-forming species, *Gymnodinium breve*, which have caused red tides in the GOM in the past (Tester and Steidinger 1997). As these tend to be mostly surface dwellers, however, it is more likely that these bottom sequences either originated from dinoflagellate resting cysts, of which *Gymnodinium* is capable of forming (Morquecho and Lechuga-Deveze 2003), or from a nonblooming, heterotrophic species. Nitrogen availability in the Atchafalaya River Plume and the Mississippi River Plume causes such an increase in the phytoplankton community, that the resulting flux of phytoplankton to bottom waters provides an abundant carbon source for bacteria (Green et al. 2006; Diaz and Rosenberg 2008). This, in turn, provides ample prey for such heterotrophic dinoflagellates.

Clustering of MALV I hypoxic OTUs (11 OTUs) with BLAST results varied geographically, and MALV II hypoxic OTUs from station 8C formed a unique cluster (GOM II) at the bottom of the MALV II clade. To date, MALV I and II groups represent the largest portion of published dinoflagellate sequences from marine ecosystems. Group II MALV sequences have previously been found to be more abundant in clone libraries from the water column, and are all from the order Syndiniales (Loeblich 1976). Further accurate affiliations are still difficult to place (Countway et al. 2007; Guillou et al. 2008), apart from the knowledge that Syndiniales is a known obligate parasite, able to infect a variety of hosts including radiolarians. The presence of *Polycystinea* in the bottom waters of station 10B and AB5 therefore suggests a possible symbiosis here. Group I MALV lineages have been found more frequently within anoxic/hypoxic environments, hydrothermal vents, and sediments (Guillou et al. 2008). All group I sequences in this study came from bottom-water samples, consistent with Guillou's study. Microbial marine parasites, Syndiniales included, are not well studied, but their presence in this study and others focusing on low-oxygen marine habitats (Edgcomb et al. 2011) could signify a significant energy shift in trophic links in hypoxic to anoxic waters.

Eight hypoxic sequences clustered with the stramenopiles, half of which clustered with the diatoms such as *Cyclotella*, *Skeletonema*, and *Thalassiosira* species, all of whom are indicators of high eutrophication and high salinity (Weckström and Juggins 2005), consistent with these waters. The remaining four hypoxic stramenopile OTUs were harder to place phylogenetically. These novel marine stramenopile (MAST) species tend to branch at the base of the stramenopile tree, similarly to the novel MALV clades. The few studies analyzing the phylogeny of these sequences to date have found MAST sequences to occupy a wide variety of habitats. The clusters found in this study affiliated with MAST 3 and 7 clusters, which are found in almost all open sea and coastal areas (Massana et al. 2004; Not et al. 2009). MAST 3 sequences, however, have more recently been reported in anoxic and low-oxygen habitats (Wylezich and Jurgens 2011). Such novel heterotrophic cells are mostly phagotrophic free-living flagellates (Fenchel 1986; Arndt et al. 2000). As such, they are the primary grazers of marine microbes and play a vital role, acting as a link between picoplankton and larger phagotrophs and by recycling inorganic nutrients (Massana et al. 2002). More surveys need to be done, but the presence of such organisms in hypoxic waters could be essential for proper ecosystem functioning here.

A few 8C_B clones clustered weakly with the Cercozoa, bottom-dwelling mixotrophic picoeukaryotes that typically feed upon bacteria, fungi, algae, and other protozoa. Phylogenetic placement of these organisms is still not clear, however (Bass and Cavalier-Smith 2004), and so corresponding trophic contribution of this class cannot yet be ascertained. 8CB04 in particular clustered well with a clone from the sediments of Sagami Bay where the authors were not able to associate it to any known taxa (Takishita et al. 2007). Cercozoa have been found in low numbers only in other low-oxygen environments (Stoeck et al. 2010; Edgcomb et al. 2011; Orsi et al. 2012), also consistent with this study.

The remaining clades belong to autotrophic picoeukaryotes. Given the low to no available light in the bottom layers of the study site, these two OTUs can most likely be attributed to newly fallen dead algae from the surface layers.

Although oxygen levels in the bottom waters of station AB5 are not yet hypoxic, its dissolved oxygen value of 2.8 mg O_2_ L^−1^ is approaching low enough levels to represent a possible oxic–hypoxic interface where a species composition shift is starting to take place.

OTUs here almost exclusively clustered with the dinoflagellates, MALV groups I and II. The prominence of MALV I and II OTUs in this sampling site, where waters are in the process of becoming hypoxic could provide a glimpse of the shift of trophic energy from grazing to parasitism as oxygen concentrations decline. The oxygen level here is just high enough for the small parasitic dinoflagellates to thrive, but just low enough to keep larger predators at bay.

### Surface samples

When compared with hypoxic sites, the surface samples in this study show less of a trend. Clearly, surface waters at station AB5 are influenced more by the DOM and nutrients from the Mississippi Plume than by oxygen concentrations (Bianchi et al. 2011). Unlike the other stations, there is a dominance of a large amount of diatoms here, accompanied by a few Prasinophytes and Cryptophytes. All are autotrophic classes, which matches the higher <10 chl *a* fraction here. Diatoms, as well as Prasinophytes and Cryptophytes are documented as preferable prey for both mixotrophic and heterotrophic dinoflagellates (Jeong et al. 2010).

The surface waters of the remaining two stations bear a resemblance to each other. More dinoflagellates are found here, most of which affiliate with the autotrophic clades. Many of these BLAST search results are bloom-forming species, perhaps remnants of a previous bloom. There are three clones at the bottom of the dinoflagellate tree, however, that do not affiliate with any known Genbank species. Bootstrap values here are also very low, so placement here is hard without morphological details. Most likely they are a relative of the MALV clades (Diez et al. 2001; López-García et al. 2001; Moon-van der Staay et al. 2001; Moreira and López-García 2002). Such tree topology has been described in several recent studies (Massana et al. 2004; Chambouvet et al. 2011; Wylezich and Jurgens 2011). It is interesting to note that although surface samples contain the expected autotrophic classes, there are one or two parasitic OTUs among them. The parasitic cells present in surface waters could be evidence of the buffering community discussed earlier. Again, more thorough throughput sequencing methods should confirm this theory.

A few unique clusters appeared in the surface samples. The GOM I cluster affiliated closely with Adenoides. The unique cluster of 8C surface samples (39, 22, 13) are identified as novel stramenopiles, most likely part of a diatom clade, given the strong bootstrap support here. These are most likely known species not yet available in the Genbank database. The presence of such clusters proves the need for more thorough sampling of picoeukaryotes.

The species composition in each clone library shifted significantly with oxygen concentration (Fig. 5) and was dominated by dinoflagellates with the exception of site AB5_S. This may be explained by the high number of dinospores that are produced during the dinoflagellate life cycle. Nevertheless, these spores are short lived upon production (Coats and Park 2002) and so their presence suggests a constant production despite hypoxic conditions. The strong clustering of novel syndiniales clones suggests species-specific functioning in this habitat. Other than their known parasitic affiliations, morphology of these organisms needs to be ascertained before accurately describing their ecological function.

Parasitism at this level in the marine environment is not well studied. The few studies to date, however, have identified dinoflagellate parasites able to infect a range of species including ciliates, invertebrates, vertebrates, free-living dinoflagellates, and even other parasitic dinoflagellates. More recently *Amoebophrya* sp., a known parasitic dinoflagellate have been found exploiting dinoflagellate blooms in enriched coastal environments, where the percentage of hosts infected have been found to exceed 80% (Coats 1999; Johansson and Coats 2002). This same species was found closely related to many of the clones in this study, all exhibiting host relationships with free-living dinoflagellates, some of which are known bloom-forming species (*Alexandrium* sp., *Karlodinium* sp., *Prorocentrum* sp.). The literature confirms that *Amoebophrya* is one of the few parasitic dinoflagellates without host preference, other than a tendency to mostly target free-living dinoflagellates (Coats 1999; Coats and Park 2002). Successful infection of a host by such a parasite consists of an infective dispersal stage, the dinospore, an intracellular growth stage, the trophont, and an extracellular reproductive stage, the vermiform. At maturity, the vermiform ruptures though the host cell, resulting in hundreds to thousands of new dinospores (Coats and Bockstahler 1994). The presence and survival of such a lifecycle in hypoxic waters such as the GOM could explain the large number of such parasitic clones in this study. The genetic diversity of *Amoebophrya* sp. is also incredibly high, consisting of 44 known separate clusters to date, mostly by environmental sequences (Guillou et al. 2008; Chambouvet et al. 2011). More research is needed in order to properly ascertain how host specific each clade really is, and it will be of critical importance to study whether hypoxic conditions stimulate the maturity of the parasitic organism and enhance the production of dinospore.

In conclusion, this study has revealed a high genetic diversity of picoeukaryotes in hypoxic waters of the GOM along with a notable shift in the microbial carbon pathway from typical grazing to parasitism. The survival of such dinoflagellate species in hypoxic waters is not well documented to date and is a significant shift worthy of more research. The evolution of today's high-throughput methods will no doubt shed some light on the true role these novel species play in hypoxic habitats. The lack of protist predators in such waters combined with an increase in bacterial abundance and diversity will more than likely provide a refuge to smaller, more tolerable microbial species such as those found in this study.
